# Surgical Strategies and Challenges in Scheuermann’s Kyphosis: A Comprehensive Review

**DOI:** 10.3390/jcm14124276

**Published:** 2025-06-16

**Authors:** Angelos Kaspiris, Ioannis Spyrou, Fotios Panagopoulos, Vasileios Marougklianis, Periklis Pelantis, Michail Vavourakis, Evangelos Sakellariou, Ioanna Lianou, Dimitrios Ntourantonis, Thomas Repantis, Elias S. Vasiliadis, Spiros G. Pneumaticos

**Affiliations:** 1 Third Department of Orthopaedic Surgery, School of Medicine, National and Kapodistrian University of Athens, “KAT” General Hospital, Nikis 2, 14561 Athens, Greece; giannisspyrou1994@gmail.com (I.S.); billma@med.uoa.gr (V.M.); pelantisperiklis@gmail.com (P.P.); michail.vavourakis@hotmail.com (M.V.); vagossak@hotmail.com (E.S.); ilivasil@med.uoa.gr (E.S.V.); spirospneumaticos@gmail.com (S.G.P.); 2 Department of Orthopedic Surgery, “Rion” University Hospital, School of Health Sciences, University of Patras, 26504 Patras, Greece; panfo97@gmail.com; 3 Department of Orthopaedics, “Agios Andreas” General Hospital of Patras-NHS, 26224 Patras, Greece; jolianou@hotmail.com (I.L.); d_douradonis@yahoo.gr (D.N.); tomrep@gmail.com (T.R.)

**Keywords:** Scheuermann’s kyphosis, surgical indications, operative approaches, instrumentation techniques, infections, neurological complications, proximal junctional kyphosis

## Abstract

**Background**: Scheuermann’s kyphosis (SK) is characterized by anterior wedging of >5 degrees at three or more contiguous vertebrae associated with severe back pain and cosmetic disfigurement. Different surgical interventions have been applied for SK correction, but the optimal operational treatment remains controversial. **Objectives:** The aim of our study is to analyze all the current indications for the surgical correction of SK, as well as to describe the instrumentation methods and techniques in order to detect the ideal operational management and accompanied complications. **Methods**: This comprehensive review investigates the up-to-date surgical indications and approaches for SK, the current trends, and the associated postoperative functional outcomes. A detailed search of PubMed, Web of Science and Google Scholar databases in English literature was performed for articles during the last 20 years. Additional criteria were peer-reviewed original studies that provided the type of interventions for SK and the clinical outcomes. **Results**: Thirty studies that met our induction criteria were analyzed. The up-to-date surgical indications such as back pain, failure of conservative treatment, progression of deformity, and neurological complications were described. Anterior (AO) and posterior-only (PO), and combined anterior–posterior (AP) approaches and instrumentation techniques were outlined. The most common side effects of the above interventions were hardware failure, loss of correction and Proximal Junctional Kyphosis. Contrariwise, in PO, reduced blood loss and operational duration was noted. **Conclusions**: Although the published studies reported contradictory results of the effectiveness of the various techniques applied for SK treatment, the PO fusion was correlated with a decreased rate of complications that resulted in its current increase in popularity.

## 1. Introduction

Scheuermann’s disease, first described by Danish orthopaedist and radiologist Holger Werfel Scheuermann in 1920, is a rigid developmental kyphotic deformity characterized by a Cobb angle of more than 45°, typically with the apex located in the thoracic and less commonly in the thoracolumbar spine (atypical SK) [1]. It is the most common growth deformity affecting the sagittal plane of the spine [2]. Diagnostic criteria were further refined by Sørensen in 1964, who defined the condition by the presence of at least three adjacent vertebrae, each wedged by a minimum of 5° each [3]. Contrariwise, the atypical form of SK, whose prevalence is 2.8%, is defined by the lack of the thoracic kyphosis, absence of vertebral wedging and location of apex kyphosis at the thoracolumbar junction (T11–T12) [4].

Although the incidence of Scheuermann’s disease ranges from 0.4 to 10%, its etiology remains unclear, with heritability estimated at approximately 74% [5,6]. Even though the exact cause of SK is unknown and genetical factors have been implicated in its pathophysiology, excessive mechanical loading and endplate defects also contribute to SK progression [7]. It is postulated that disrupted anterior endochondral ossification due to increased pressure leads to the characteristic wedge-shaped vertebrae [7]. The condition most commonly affects adolescents aged 12 to 16 years, with a near-equal distribution between males and females. It typically presents as a rigid kyphotic deformity, usually accompanied by hamstring tightness [6]. Scheuermann’s disease should be differentiated from the postural kyphosis of adolescence, taking into account the neurological examination and the assessment of joint range of motion [8]. Moreover, SK has effects on rib cage morphology [9]. Measurements of the shape of vertebral bodies, angle of transverse processes, and sternum and rib anatomy revealed longer and flatter ribs and increased anteroposterior diameter in the SK group compared with control group, which may result in respiratory complications in patients suffering from severe form of SK [9]. Diagnostic protocols for SK include radiological imaging techniques such as conventional plain radiographs, computer tomography (CT-scan) or Magnetic Resonance Images (MRI) in order to reveal Schmorl’s nodes, or disc abnormalities and vertebral Modic changes [9]. Recently, rasterstereography, which is a non-invasive method of 3D surface topography applied to measure the height and curvature of SK, demonstrates increased validity and reliability compared with the X-rays [10].

Treatment of Scheuermann’s disease aims to reduce pressure on the anterior vertebral endplates to alleviate pain [2]. Conservative treatment with physiotherapy or support measures can be effective for curve angles less than 60°. For curves between 60 and 80°, bracing may be indicated, particularly when applied during skeletal growth to halt curve progression [11,12]. While most cases can be effectively treated with conservative measures, kyphotic curves greater than 70–75° with severe pain not responding to non-surgical measures and/or respiratory restrictions require surgical interventions [13,14].

Surgical strategies for Scheuermann’s kyphosis aim to achieve three key objectives: the release of spinal lesions, correction of at least 50% of the kyphotic deformity, and instrumented spondylodesis [15]. These outcomes can be achieved either via a posterior-only approach or through a combined anterior–posterior procedure, which incorporates anterior release followed by posterior instrumentation [16,17]. However, this method is associated with higher complication rates, increased operative time and greater blood loss [18,19]. Although combined approaches were previously favoured, more recent techniques—including dorsal column shortening with osteotomies and dorsal instrumentation—have demonstrated comparable results [20]. Comparative studies on surgical approach selection remain inconclusive. While some report superior patient satisfaction with combined approaches, others demonstrate improved fusion rates and lower complication rates with the dorsal technique [21,22].

Τhe comparison of postoperative side effects between techniques has not been clearly documented yet [19]. Given the lack of adequate reports clarifying the optimal surgical approach and fusion level, and the ongoing research on risk factors affecting operation results, the aim of our study was to analyze all current indications for the surgical management of SK, the interventions applied for its treatment and the associated complications and outcomes.

## 2. Materials and Methods

A systematic computer-based comprehensive literature review search was performed on 10 March 2025 in the following databases: PubMed (1947 to present) and Web of Science (1900 to present). The research methodology used a combination of the following terms: “Scheuermann’s kyphosis [All Fields]”, “surgical techniques [All Fields]”, “surgical indications [All Fields]” and “outcomes and complications [All Fields]”. The electronic literature search was conducted independently by two authors (AK, SGP) and an experienced librarian. Moreover, the above two senior authors (AK and SGP) independently screened the titles and abstracts to identify relevant studies of clinical outcomes and complications after surgical interventions in patients suffering from Scheuermann’s kyphosis. If there was a disagreement between them, the final decision was made by the senior author (SGP).

Qualitative studies that analyzed the clinical outcome in patients after the surgical treatment of Scheuermann’s kyphosis were identified. Only full-text articles were eligible for inclusion. Additional inclusion criteria included (a) studies written in English (b) comparative studies assessing the application of different techniques and instrumentation methodologies for the treatment of Scheuermann’s kyphosis, (c) surveys concerning the surgical approaches and (d) data on the outcome should have been clearly given to each patient. Publication date limitations were not set.

Surveys written in a language other than English were excluded. Case reports, reviews, letters to the editor, expert opinions and articles with insufficient details about the type of intervention, the clinical outcome and surveys with non-obtainable data were excluded. Research based only on in vitro or in vivo animal models’ results was also excluded.

This review follows the methodology of a narrative review, providing a conscientious synthesis of the available literature. Moreover, the certain limitations of the included studies, such as the availability and accessibility of the articles, and potential publication bias were clearly reported. Finally, thirty studies (Table 1) met the qualitative criteria and were included in our analysis [16,18,21,22,23,24,25,26,27,28,29,30,31,32,33,34,35,36,37,38,39,40,41,42,43,44,45,46,47,48].

## 3. Epidemiology and Definition

### 3.1. Prevalence

Data on SK prevalence varies in the literature, with estimates ranging from 0.4 to 10% of the general population [49,50]. In 1951, Wassmann investigated Danish army recruit reports that included young men rejected from service because of diseases of the ‘back, thorax and pelvis’ [51]. He conservatively estimated that 4–5% of adult Danish men suffered from SK; this, however, was prone to bias, as mild cases may have evaded diagnosis and the SK rates attributed to the aforementioned category were based on a rough estimate [51]. In a 2015 multi-centre study of more than 10,000 patients (ages 50–74) across Europe, Armbrecht et al. reported an overall prevalence of 8% [51]. This, however, varied across individual countries [52]. In another 2018 study, 454 patients aged 15–40 years old undergoing chest radiographs for unconnected reasons were studied for the presence of consecutive kyphotic vertebrae or kyphosis of over 40 degrees. The authors concluded on a SK prevalence of 2.2%. Cases of the rarer type-2 thoracolumbar kyphosis may have been missed in this study though [53]. Scoles et al. in 1991, evaluating 1384 cadaveric spines, reported a 7.4% prevalence of SK [7]. Makurthou et al. in an observational study in the Netherlands discovered radiographic features of SK in 4% of the population of studied adults aged 45 years or older [50].

A recent study from Brazil investigated the prevalence of atypical SK in asymptomatic patients undergoing abdominal CT scans. The authors found a prevalence of 2.8% [4] with equal distribution between males and females [49]. Atypical SK typically affects adolescent males active in sports and heavy lifting; it does not commonly present with significant deformity nor is it progressive, and it often resolves with conservative measures [54].

### 3.2. Diagnostic Criteria

**Figure 1:**
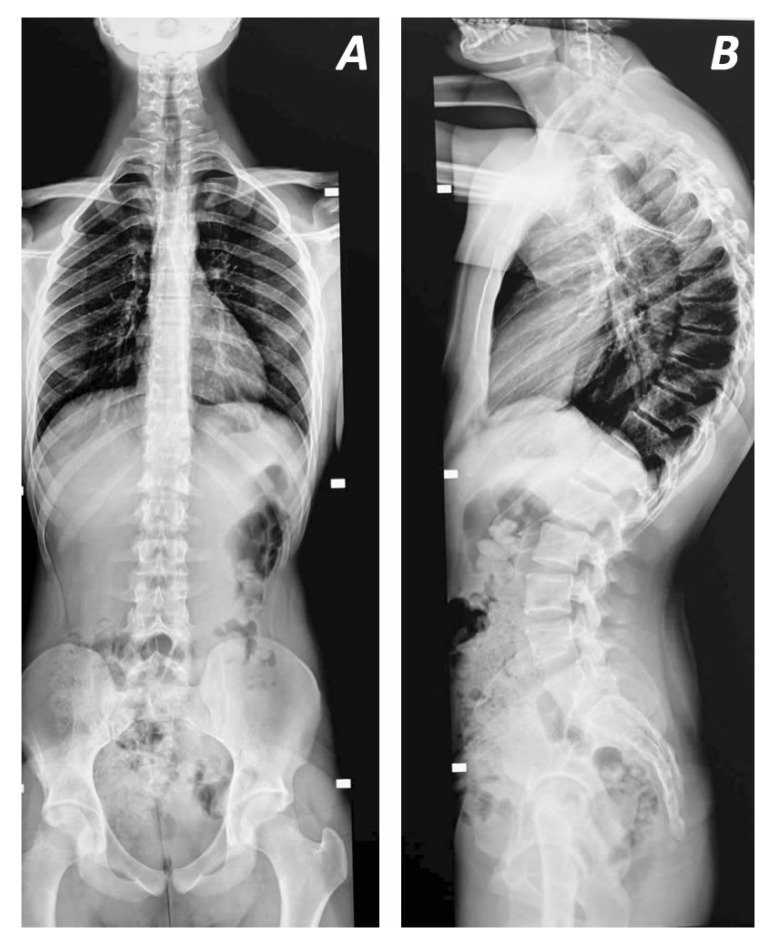
Anteriorposterior (**A**) and lateral (**B**) views of characteristic kyphotic deformity of 77° between T5 and L1 with apex at T9 (**B**). Note the vertebral wedging at these spinal locations (**B**).

The diagnostic criteria of SK were first proposed by Sorensen in 1964, defining the condition by the presence of at least 3 adjacent vertebrae, each wedged by at least 5° [3]. Sachs modified these criteria and proposed that the diagnosis could be established with at least one wedged vertebra of ≥5°, in combination with thoracic kyphosis exceeding 45° [55] (Figure 1). Associated findings of SK are Schmorl’s nodes, increased anteroposterior diameter of the affected thoracic vertebral bodies, decreased intervertebral disc spaces, and irregular endplates [7,56]. However, these are not always encountered in atypical SK. German literature also references the rare Edgren–Vaino sign, namely the proliferation of bone opposite a Schmorl node, as a pathognomonic finding [52]. MRI findings also suggest accelerated thoracolumbar disc degeneration, even in young patients [57]. Notably, a 2017 study by Lonner et al. reported that preoperative MRI findings led to changes in surgical planning based on these findings [58]. In this prospective multicentre research, the correlated deformities such as (a) presence of spinal cord abnormalities, like syrinx or low lying conus, (b) location and number of vertebral wedging, Schmorl nodes or posterior disc herniation, (c) existence of spondylolysis, (d) degree of spinal stenosis and (e) segmentation deformities, were also examined, indicating the necessity for including further spinal parameters in the severity of final diagnosis. Although the results of the MRI altered the operative plans in 5% of the cases, the overall rate of postoperative neurological complications remained unchanged [58].

## 4. Operative Management

### 4.1. Surgical Approaches

Surgical indications remain controversial due to the limited evidence available regarding the natural history and progression of the disease.

For mild SK, most available data suggest a benign clinical course. Ristolainen et al. published on a long-term follow-up study of untreated SK (mean kyphosis 45°) patients, with a mean of 37 years [59]. A higher risk for back pain and difficulty with daily living activities was reported by patients compared to the control group [59]. However, follow-up radiographic data were not available. These results were also in line with an earlier long-term study by Murray et al., who found that SK patients had more intense back pain than controls [60]. This, however, did not interfere with daily activities, the use of pain medication, days absent from work, or self-perceived health. Pulmonary function was affected only in cases with kyphosis angles exceeding 100°. Garrido et al. followed-up patients with conservatively treated SK (mean initial kyphosis 66°) for a mean of 27 years [61]. Patients reported reduced QoL scores compared to the general population. A mean kyphosis progression rate of 0.45° per year was also documented. In another study for mild untreated SK (mean kyphosis 46°), with a mean follow-up of 46 years, a radiographic progression of 14° was discovered; likewise, an increase in lumbar lordosis was noted [61]. This, however, did not correlate with symptoms.

Surgical treatment is recommended for patients with a fixed or progressive deformity unresponsive to bracing, particularly in skeletally mature adults with kyphotic curves measuring 60–80°, or in adolescents with curves exceeding 75° [37,62,63,64,65]. However, results from various centres indicate that surgical indications on curve magnitude vary among surgeons, with the mean curve of patients surgically treated estimated at 70°, versus 73° for the conservatively managed group [49]. Thoracic myelopathy or neurological deficits are absolute indications for operative treatment, while relative indications include severe pain and notable deformities, including kyphosis of the upper lumbar spine, thoracic kyphosis of above 75° or kyphosis of above 65° located in the thoracolumbar junction [15,66].

Other factors prompting surgical consideration in juvenile kyphosis are painful kyphotic curves unresponsive to conservative treatment, neurological compromise, psychological concerns associated with spinal deformity—such as impaired self-esteem and body image—in growing adolescents and pain refractory to conservative treatment [11,63,64]. A more recent consideration emerging in the literature regards lumbar lordosis. Haselhuhn et al. proposed that excessive compensatory lumbar hyperlordosis in patients with SK may justify surgical intervention, as it may accelerate degenerative changes in the hyperextended segments of the lumbar spine [67]. However, further studies are needed to investigate this.

Surgical treatment of Scheuermann’s kyphosis has changed since the initial introduction of the Harrington rod. The use of segmental instrumentation and posterior-only approaches based on pedicle screws, first introduced by Roy-Camille, enabling surgeons to correct a deformity in multiple planes, has consolidated in modern spine surgery [68]. There are two surgical management strategies used for Scheuermann’s kyphosis; combined anterior release and posterior fusion, and posterior-only fusion with different anchors [64]. Posterior-only was initially used for the correction of Scheuermann kyphosis, as described by Bradford [48]. This technique is considered effective for the treatment of Scheuermann’s kyphosis, whereas a combined anterior–posterior approach is generally recommended for severe and rigid deformities, with careful consideration of the potential complications.

The factors that must be evaluated are age, curve rigidity and any possible complications according to the patient’s profile [12]. In young patients whose skeleton is still growing, posterior-only fusion is preferable, while the combined approach is the treatment of choice for adults with more rigid curves [11,69]. The anterior–posterior fusion technique offers superior correction outcomes in patients with significant and rigid deformities, demonstrating improved correction rates, enhanced Bending Brace Correction Index (BBCI), and greater gains in spinal height [12,70]. Combined fusion has been associated with a lower incidence of junctional failure, particularly when utilizing instrumentation systems such as Cotrel–Dubousset instrumentation or Luque rods [45]. Consequently, combined fusion was considered the gold standard for SK. However, with the advancement of new techniques like Ponte’s osteotomy and instrumentation material with multi-segmental posterior pedicle screws, there is a shift towards posterior-only fusion [22]. The Ponte osteotomy entails resection of the facet joints, the laminae and ligamentum flavum in the involved vertebrae, and shortens the posterior spinal column in order to reduce kyphosis, while retaining the anterior ligamentous structures [71]. Although it was originally developed in the 1980s to address thoracic hyperkyphosis, recent studies demonstrated that it is a safe and effective intervention either in the correction of Idiopathic Scoliotic (IS) deformities or SK [49]. Moreover, Ponte’s osteotomies in SK correction are associated with remarkably reduced blood loss and intraoperative time compared with IS [72].

**Figure 2:**
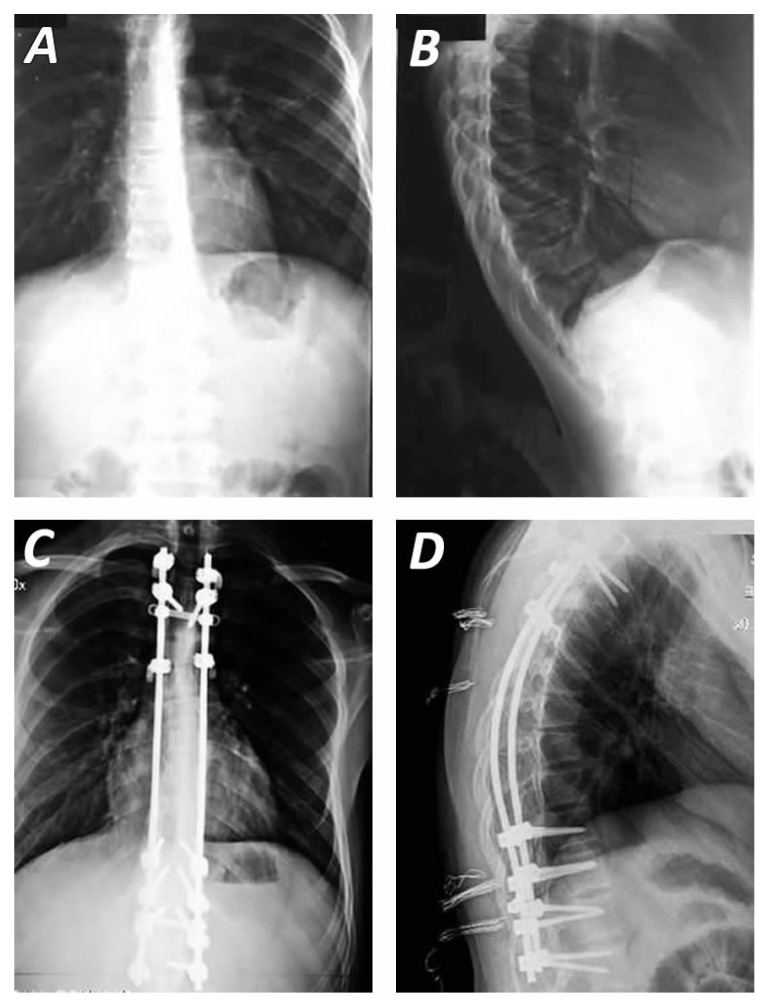
Pre-(**A**,**B**) and postoperative (**C**,**D**) radiographs of an 18-year-old male patient with Scheuermann’s kyphosis, depicting thoracic kyphosis and the correction of deformity. Posterior spinal fusion (T3–L2) was performed with use of pedicle screws (T4, T11, T12, L1, L2) and transverse process hooks (T3, T6) connected to dual pre-curved compression rods accompanied with two cross-links. Facet osteotomies (T6, T7, T8, T9, T10) and spinous process removal (T6, T7, T8, T9, T10) were necessary for curvature correction and fusion along with use of allograft (**C**,**D**). The preoperative kyphosis was 79° degrees extended from T3 to L2 (**B**), while after the fusion of T3 to L2 the postoperative angle was 38–40° degrees (**D**).

The decision between combined anterior–posterior fusion and posterior-only fusion in the surgical management of Scheuermann’s kyphosis remains a topic of ongoing debate [30]. Numerous comparative studies have attempted to resolve this controversy. A systematic review and meta-analysis by Yun et al. found that posterior-only fusion was associated with reduced blood loss, shorter operative times, and lower incidences of proximal and distal junctional kyphosis (PJK/DJK), as well as decreased rates of revision surgery, when compared to the combined approach [17]. Conversely, McDonnell reported that the sagittal vertical axis was the only spinopelvic variant favouring the dual approach [70]. However, it is crucial to acknowledge that in patients with rigid curves where the anterior longitudinal ligament is intact with the posterior-only technique, the posterior instrumentation would be subjected to constant tensile stress, leading to pseudarthrosis and increased risk of implant failure [64]. A meta-analysis by Li including 586 patients, demonstrated that although posterior-only fusion (Figure 2) had advantages in terms of operative time and blood loss, both surgical strategies were comparably effective in achieving deformity correction in Scheuermann’s kyphosis [19].

Both surgical procedures have been used for the treatment of Scheuermann’s kyphosis for decades and their instrumentation has greatly evolved. Anterior release was historically considered integral to achieving optimal correction, as it allowed for greater angular correction, improved mechanical support during the fusion process, and enhanced structural rigidity with the use of structural grafts. This procedure typically involved an apical anterior spinal release, followed by posterior instrumentation and fusion [13]. Video-assisted thoracoscopy (VATS) could be used as an alternative anterior approach other than the usually performed thoracotomy [39]. Animal studies have shown that VATS release can achieve equal spine flexibility with thoracotomy [73].

### 4.2. Surgical Techniques and Instrumentation Methods

Predominantly, four instrumentation techniques have been used for SK correction.

The Harrington compression system, which consists of two 1/4-inch threaded Harrington compression rods with three proximal hooks anchored to the thoracic transverse processes and three sublaminar hooks distally on the lumbar vertebrae [63,74]. Originally designed to address severe scoliotic deformity, this technique underwent several modifications over the years to increase instrumentation strength and maintain the correction achieved, as at first it included one distraction bar applied without fusion as an internal brace [67,74]. This was secured to the laminae or the transverse processes. Postoperatively, patients were required to wear external braces. However, these systems were frequently associated with postoperative loss of correction and hardware failure [68,75].

In 1973, Luque developed and published a novel technique, using sublaminar wires and rods [76]. By combining facetectomies, ligamentous release and partial removal of ligamentum flavum, the wires are then tightened to the prebent rods to reduce deformity. This is performed at each level, achieving rigid fixation [77].

The Cotrel–Dubousset (CD) technique, introduced in the 1980s, combines two rods and multiple segmental bone hooks. Transverse rods may also be used to augment construct stability. This technique has also been adopted for scoliosis correction [78]. It uses a two-hook configuration at the cephalic end, where rods are introduced. A cantilever maneuver is then performed to guide the rods into the hooks of a single claw construct at the caudal end of the deformity, enclosing and correcting the curvature [63,79,80,81]. Notably, this technique reduces dependency on postoperative thoracolumbar bracing [78].

Both the Cotrel–Dubousset and Louque procedures have been extensively used in the surgical management of spinal deformities. However, both are associated with the development of junctional kyphosis [43].

**Figure 3:**
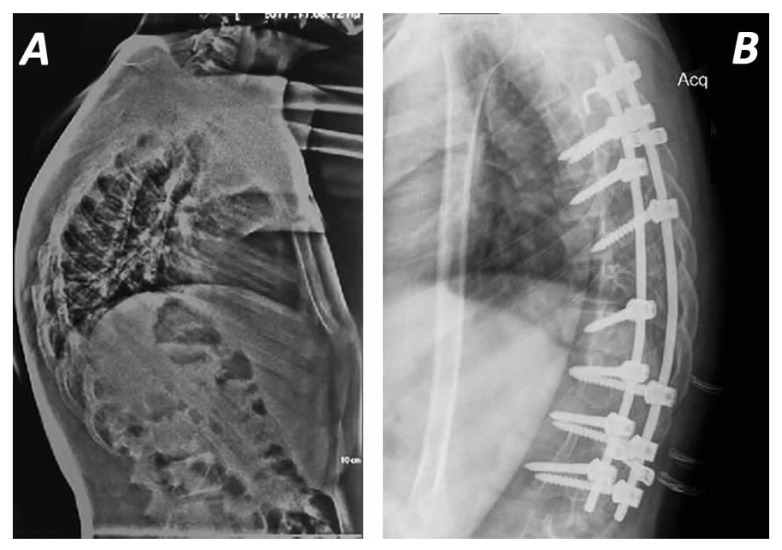
Pre- (**A**) and postoperative (**B**) radiographs of a 16-year-old male with Scheuermann’s disease. Posterior spinal fusion (T5–L1) was achieved with use of pedicle screws (T6, T7, T8, T10, T11, T12, L1), transverse process hooks (T5) dual (compression pre-curved) rod system and two cross-links. Fusion was enhanced with fresh frozen allograft (**B**). It must be highlighted that proximal bone hooks to prevent the Proximal Junctional Kyphosis (PJK) does not show any statistically significant difference compared to purely pedicle-screw-based constructs. The preoperative kyphotic deformity was 85° degrees extended from T2 to L2 (**A**), while after the spinal fusion between T2 to L2 was limited to 37° degrees (**B**).

These techniques are enhanced by new surgical modifications with multi-segmental posterior pedicle screws and Ponte’s osteotomy, in which the posterior ligaments (supraspinous, intra-spinous ligaments and ligamentum flavum) and the facet joints are removed, and correction is performed through the disc space (Figure 3) [12,71].

A fourth technique, often employed in conjunction with posterior instrumentation, involves anterior release of the shortened anterior longitudinal ligament, combined with discectomy on the curve, and the placing of grafts under compression conditions [20,62]. To prevent curve recurrence and promote robust fusion, large-diameter, stiff rods, along with iliac crest bone grafting, may be utilized across all techniques [13].

## 5. Outcomes

Given the absence of universally accepted surgical indications, treatment must be individualized for each patient. Nevertheless, when compared to conservative management, surgical intervention offers more reliable deformity correction.

In 1975, Bradford published the first case of a series of patients with Scheuermann kyphosis treated operatively via a posterior approach using Harrington rods. A significant drawback observed was loss of correction with the posterior approach alone [17,48]. A staged approach—involving anterior release and fusion at the apex followed by posterior instrumentation—was later recommended [43,57].

In 1979, Taylor et al. reported their results using posterior-only Harrington compression rods in adolescents with SK, achieving an average correction of 26 degrees [47,79]. Furthermore, the anterior longitudinal ligament was increasingly recognized as a key contributor to the deformity [47,82]. Additionally, Birnbaum et al., in a cadaveric study, demonstrated that isolated anterior longitudinal ligament transection led to a 4-degree Cobb angle change per released segment, with an additional improvement of 2 degrees if followed by osteodiscectomy at the corresponding level [83]. This technique, which sufficiently mobilizes the anterior spinal column, may be performed via minimally invasive methods, thereby reducing the morbidity associated with formal open procedures. In 1986, Speck and Chopin recommended a combined anterior–posterior approach for skeletally mature patients (Risser sign stage 4 or 5), whereas a posterior-only approach was considered appropriate for skeletally immature individuals, with lower risk of significant loss of correction [79]. Many authors suggest combined-approach surgery for severe, rigid curves [46,63].

Lowe and Kasten reported excellent results using a staged approach with CD instrumentation, achieving correction of severe kyphotic deformities (mean of 85°) with minimal correction loss (average of 4°) postoperatively [43]. Similar results are corroborated in the literature [33].

More recent reports advocate for the posterior-only approach, facilitated by advances in spinal instrumentation techniques [40,84]. Moreover, all-pedicle screw instrumentation has largely eliminated the need for anterior release, allowing stable fixation and sufficient reduction with aggressive osteotomies [36]. Similar correction rates and loss of correction are seen with both approaches, even in the long term [31,38,40,69]. In a study involving patients with a mean kyphosis of 71 degrees, Sturm et al. suggested that posterior-only instrumentation could effectively counteract the tensile forces of the anterior longitudinal ligament and anterior spinal column, provided no anterior bony bridging is present [44]. This technique thereby avoids the physiological burden and potential complications associated with a second surgical procedure. A 2017 systematic review by Yun et al. showed similar degrees of correction loss between anterior–posterior and posterior-only techniques, with posterior-only approaches demonstrating improved correction maintenance in the long term [17]. Pedicle screw constructs in posterior-only-treated patients with low-density screws placed (54–69% of available pedicles) are equally safe and effective, with high-density (100% of available pedicles) constructs in SK treatment, while being more cost-effective and reducing the chances of the screw malpositioning-related complications associated with the latter [32]. Additionally, in immature patients, the application of pedicle screws and posterior vertebral tethering resulted in gradual correction of thoracic kyphotic deformity and maintained the mobility of tethered segments [26]. The above findings were accompanied by significant improvements in the thoracic and lumbar lordosis, vertebral wedge angles, spinopelvic parameters and spinal motion without development of neurological or infectious complications [26]. A 2005 review by Arlet suggested that surgical treatment in SK is highly effective in reducing pain and providing satisfaction rates of up to 96% [13].

According to a retrospective study of 693 patients with surgically treated Scheuermann’s kyphosis, reported by the Scoliosis Research Society, the overall complication rate was 14% [85].The most common complications of both posterior-only and combined anterior with posterior approach techniques include junctional kyphosis (18.4% PJK, 12.7% DJK), wound infections (15%), neurologic complications (2%), union complications (4%), hardware failure or hook pull-out (8%), and loss of correction, painful bursitis over prominent hardware (0.5%), deep vein thrombosis and pulmonary embolism (1%), cardiopulmonary problems (1%), pulmonary complications such as pneumothorax (2%), gastrointestinal disorders (0.5%), postoperative back pain or sciatica and cervical pain (19%). These results are presented in Table 1. Pulmonary function is adversely affected immediately postoperatively in patients undergoing open thoracotomy for anterior spinal fusion, but returns to 95% of baseline within 2 years of follow-up [86]. This should be taken into consideration when selecting surgical candidates.

Distal and Proximal Junctional Kyphosis (DJK-PJK), which is defined as the development of a kyphotic angulation over 10 degrees below or above (accordingly) a fusion construct, has been described as a complication of the treatment of Scheuermann kyphosis accounting to the 5–46% and 13–38% cases that underwent spinal fusion, respectively [80]. In a study evaluating combined anterior and posterior approaches, Lowe et al. suggested that correction exceeding 50% of the initial kyphotic angle predisposes patients to the development of PJK [43]. This has been disputed by other authors [87]. The upper threshold of thoracic kyphosis, often cited as 40–50 degrees (as proposed by Bernhardt and Bridwell), has been recommended as a correction target to prevent sagittal imbalance and reduce the risk of PJK [31,40,88]. Yuan et al. reported a higher incidence of PJK in patients with preoperative sagittal imbalance, defined by a C7 plumb line–sacrum distance greater than 5 cm [87]. However, it remained unclear whether a short proximal fusion level was the direct cause of PJK in those cases. Furthermore, including T2 or higher in the fusion reduced the PJK, which was unaffected by the inclusion of the proximal end vertebrae in the fusion construct [87]. It is generally accepted that the proximal end vertebra should be included in the fusion [43,49,65,89]. Determining this can be challenging in the upper thoracic spine; therefore, high-quality radiographs are essential. Denis et al. found an incidence of 30% PJK in their study and emphasized the importance of preserving the junctional ligamentum flavum for preventing PJK [89]. Other authors suggest adding proximal bone hooks in the constructs to prevent the PJK, although no statistically significant difference was observed in their study compared to purely pedicle-screw-based constructs [25].

This finding is also supported by the recent study of Jench et al. that reported low rates of PJK or revision operations and increased total scores of satisfaction in SRS-Questionnaires, after the application of posterior pedicle screw–dual rod system accompanied by multiple osteotomies [23]. Contrariwise, the comparative analysis between two groups of 37 patients that underwent pedicle screw and hook instrumentation fusion demonstrated mean Proximal Junctional Angles of 19° and 5° degrees, respectively, [25]. PJK may not always cause clinical or cosmetic complaints, but approximately 10% of patients may require revision surgery [88].

Although many risk factors such as older age, preoperative thoracic hyperkyphosis, increased pelvic incidence (PI), weakness of the paraspinal muscles, ligamentum deficiency or screw malposition have been implicated in the development of PJK, the effect of pre- and postoperative spinopelvic parameters are unveiled as crucial factors for this deformity [90]. The study of Gomez et al. demonstrated that preoperative thoracic kyphosis greater than 50° and postoperative pelvic tilt (PT) greater than 30° were remarkable risks factors for the development of radiographic PJK [90]. The study of Nasto et al. reported that preoperative PI and postoperative deficit of lumbar lordosis (LL) were significantly higher in the PJK groups of patients with SK, also confirming the results of the previous research [91]. Specifically, the above study displayed that the overcorrection of kyphotic deformity may result in increased reduction in LL, leading to lumbopelvic (LL-PI) mismatch and an increased rate of PJK [91]. These results are in line with the findings of recent studies that reported decreased PI values in SK patients compared to healthy controls [92,93]. Therefore, lower correction of kyphotic deformity could be desirable to prevent the lumbopelvic mismatch phenomenon [91]. This is also suggested by the findings of Ponchelet et al. who observed an increased rate of PI-LL mismatch in the PJK group. Although the above study has not displayed any association between pelvic tilt (PT) and postoperative outcomes of SK correction [94], it must be highlighted that greater postoperative PT may result in symptomatic PJK and worse patient-reported outcomes [94,95].

**Table 1:** Characteristics of the included studies.

	Study/Country	Number of Patients (N)	Age Mean and Range (in Years)	Surgical Technique	Mean Preoperative Kyphosis (in Degrees)	Mean Postoperative Kyphosis (in Degrees)	Follow up Mean and Range	Loss of Correction (in Degrees)	Complications and Outcomes (N)
**1**	Jensch et al., 2025, Germany [23]	73	27.9	Posterior-only with pedicle screw-dual rod system	75.11°	48.54°	3 years (2–6)	0.84° ± 4.99°	Increased SRS-22 functional scores Proximal Junctional Kyphosis (8)—2 revision surgeries Neurological complications (2) Wound healing disorders (2)
**2**	Lopez et al., 2024, Spain [24]	18	15.8	Hybrib bipolar posterior instrumentation with transerse hooks and polyaxial screws Schwab osteotomies (Type I and II)	73.6° (57–91°)	57.8° (18–75°)	3.6 years	N/A	Increased SRS-22 functional scores Increased VAS scores Reduced operation time (269 min), bleeding, instrumantation protrusion (0) and rate of spinal cord injury (0),infections (0) and Proximal or Distal junctional Kyphosis (3)
**3**	Baymurat et al., 2024, Turkey [25]	37	Posterior approach with pedicle screws 26.58 ± 7.55 Posterior approach with hooks fixation 25.46 ± 9.04	Posterior approach with pedicle screws (22) Posterior approach with hooks fixation (15)	Posterior approach with pedicle screws 76.55 ± 6.42° Posterior approach with hooks fixation 74.87 ± 7.82°	Posterior approach with pedicle screws 47.59 ± 6.42° Posterior approach with hooks fixation 48.26 ± 5.68°	Posterior approach with pedicle screws 94.73 ± 53.15 months Posterior approach with hooks fixation 103.07 ± 64.48 months	N/A	Increased angles of Proximal and Distal Junctional Kyphosis in Posterior approach with pedicle screws but without statistical diffrences with hooks fixation group SVA and SRS scores and spinopelvic parameters similar between the groups
**4**	Aydogan et al., 2024, Turkey [26]	10	13.1 (11–15)	Posterior approach withpedicle screws and tethering cords	73.6° (72–83°)	43.8° (41–47°)	47.6 (months) (36–60)	Further correction to 34.7° (30–35°)	Tether breakage (1) No blood transfusion Increased SRS-22 functional scores Gradual correction of the thoracic kyphosis, reverting vertebral wedging and maintaining the tethered segments‘ mobility
**5**	Debnath U et al., 2022, UK [27]	51	Posterior-only 18.5 ± 2.2, Anterior–Posterior 21.9 ± 4.8	19 Posterior-only, 32 Anterior–posterior	Posterior-only 81.4 ± 3.8°, Anterior–posterior 86.1 ± 6.0°	Posterior-only 45.1 ± 2.6°, Anterior–posterior 47.3 ± 4.8°	14 years (10–16)	N/A	Proximal Junctional Kyphosis (7) Distal Junctional Kyphosis (5)
**6**	Suominen et al., 2022, Finland [28]	22	16.7 (13–19)	Cotrel–Dubousset, Posterior-only	79° ( 75–90°)	55° (45–75°)	24 months	N/A	Deep infection (1), Proximal Junctional Kyphosis (4),
**7**	Vital et al., 2021, Portugal [29]	19	18.4	Cotrel–Dubousset, Posterior-only	83°	57°	72 months (2–12)	N/A	Proximal Junctional Kyphosis (2), Distal Junctional Kyphosis (1), Non-union (1), Infection (1)
**8**	Tsirikos A et al., 2021, UK [30]	88	15.9 (12–24.7)	86 Posterior-only Hybrid, 2 Anterior–posterior Hybrid closing wedge osteotomies (both)	94.5°	47.5°	8.5 years (2–14.9)	N/A	N/A
**9**	Riouallon G et al., 2018, France [16]	131	Anterior–posterior 23 Posterior-only 10	Cotrel–Dubousset, 123 Anterior–posterior, 8 Posterior-only	Anterior–posterior 78 ± 13°, Posterior-only 76 ± 23°	Anterior–posterior 59 ± 13°, Posterior-only 53 ± 19°	N/A	N/A	Peptic ulcer (1), Mesenteric artery collapse (2), Infections (7), Acute respiratory distress (2), Pneumonia (2), Pseudarthrosis (4), Proximal Junctional Kyphosis (5), Implant loosening (7)
**10**	Etemadifar M et al., 2015, Iran [31]	30	Anterior–posterior 20.9 ± 5.3, Posterior-only 19.3 ± 2.7	Cotrel–Dubousset, 16 Anterior–posterior, 14 Posterior-only	Anterior–posterior 83.7 ± 8.1°, Posterior-only 81.9 ± 9.4	Anterior–posterior 41.4 ± 7.7°, Posterior-only 40.1 ± 9.9°	Anterior–posterior 69.6 months (38–95), Posterior-only 45.6 (26–74)	N/A	Proximal Junctional Kyphosis (1) Distal Junctional Kyphosis (1), Pulmonary problems (2), Infection (1), Hook pull out (1)
**11**	Heiko Koller E et al., 2014, Germany [22]	166	Anterior–posterior 23.6 ± 11.4, Posterior-only 20.7 ± 10.4	Cotrel–Dubousset, 90 Anterior–posterior, 76 Posterior-only	Anterior–posterior 75.9°± 9.6°, Posterior-only 78.7 ± 10.1°	Anterior–posterior 43.4 ± 12.3°, Posterior-only 47.1 ± 11.7°	N/A	N/A	N/A
**12**	Behrbalk E et al., 2014, UK [32]	21	21 ± 7	Pedicle screws- hd vs. ld, Posterior-only	75° (61–96°)	Hd 42 ± 7°, Ld 44 ± 9°	29 months	N/A	Screw penetration into T9 disc space (1), Rod breakage and loss of correction (1), Screw loosening (2)—1 revision, Proximal Junctional Kyphosis (1)—revision, Deep infection (1)— Hardware removal-revision (1)
**13**	Temponi EF et al., 2011, Brazil [33]	28	Anterior–Posterior 19 (13–35), Posterior-only 27.3	19 Anterior–posterior thoracotomy-fusion-pedicles screws, 9 Posterior-only Smith Petersen-pedicle screws construct	Anterior–posterior 77.6°, Posterior-only 72.9°	Anterior–posterior 35.8°, Posterior-only 44.3°	Anterior–posterior 37.5 months (12.6–61.7), Posterior-only 22.8 months (31–31)	N/A	AP superficial infection (1) Screws breakage (1), Deep infection-hardware removal (1), Implant loosening—revision (1), Residual pain (4) PO seroma (1), Implant discomfort-removal (1)
**14**	Tsutsui, Shunji et al., 2011, USA [34]	22	15.1 (13–17)	Cotrel–Dubousset, 11 Anterior–posterior, 11 Posterior-only	Anterior–posterior 84.9 ± 10.2°, Posterior-only 82.7 ± 6.4°	Anterior–posterior 48.6 ± 5.7°, Posterior-only 47.9 ± 5.4°	N/A	N/A	N/A
**15**	Cho KJ et al., 2009, Korea [35]	31	18.0 ± 5.0	Cotrel–Dubousset facet osteotomy, 29 anterior–posterior, 2 Posterior-only	86.6 ± 8.5°	53.0 ± 10.4°	44 months ± 1.7	2.9° ± 6.5°	Distal Junctional Kyphosis (7), Proximal Junctional Kyphosis (3), Pseudarthrosis (2), Paresis (1), Infections (4)
**16**	Koptan WM et al., 2009, Egypt [36]	33	Posterior-only 15.9 (13.7–19), Anterior–posterior 16.8 (14.9–21.2)	Cotrel–Dubousset with sublaminar wires, 11 Posterior-only, 19 Anterior–posterior	Posterior-only 85.5° (69–102°), Anterior–posterior 79.8° (65–98°)	Posterior-only 45.1° (40–49°), Anterior–posterior 38.8° (37–45°)	53 months (24–88)	N/A	Right thigh pain (1), Infections (3) Fracture (1)
**17**	Lonner BS et al., 2007, USA [18]	78	16.7 years (9–27)	42 Anterior–posterior, 36 Posterior-only	82.6°	74.4°	33 months (24–72 years)	N/A	Pain (1), Neurogenic bladder (1), Renal failure (1), Pleural effusion (2), Pneumonic embolism (1), Proximal Junctional Kyphosis (2), Distal Junctional Kyphosis (2), Pseudarthrosis (1), Infections (2)
**18**	Geck et al., 2007, USA [37]	17	16.4 (14 to 25)	Cotrel–Dubousset, Posterior-only	75° (57° to 96°)	38° (12–50)	24 months	N/A	Infection (1), Proximal Junctional Kyphosis (1), Distal Junctional Kyphosis (1),
**19**	Lee SS et al., 2006, USA [21]	39	N/A	hook or hook/screw hybrid construct 18 Posterior-only, 21 Anterior–posterior	Posterior-only 84.4° (70–115°) Anterior–posterior 89.1° (70–104°)	Posterior-only 40.4° (30–57°), Anterior–posterior 58.0° (40–81°)	Posterior-only 31.7 months (range 24–63), Anterior–posterior 67.5 (36–146)	Posterior-only 2.0°, Anterior–posterior 2.7°	Proximal Junctional Kyphosis (2) Hook pullout (1), Distal Junctional Kyphosis (1), Paraplegia (1), Superficial infections (3)
**20**	Johnston and Charles et al., 2005, USA [38]	27	Posterior-only 6.3 (13.1–19.5), Anterior–posterior 15.6 (14.2–16.9)	Cotrel–Dubousset, 20 Posterior-only, 7 Anterior–posterior	Posterior-only 80.5° (67–97°), Anterior–posterior 79.0° (range 62–93°)	Posterior-only 38.8° (range 22–59°), Anterior–posterior 41.6° (range 34–48°)	30 months (range 24–56)	8 (range 12–8°)	N/A
**21**	Herrera-Soto et al., 2005, USA [39]	19	17.4	Cotrel–Dubousset, Anterior and Posterior	84.8° (69–105°)	43.7°	31 months (24–72)	1.6	Ulnar dysesthesia (1), Biceps weakness (1), Pull out hooks (2), Pneumothorax (2), Bursitis (1) Bleural effusion (1) None with Junctional Kyphosis
**22**	Hosman AJ et al., 2002, Netherlands [40]	33	25.8 ± 7.8	H frame, 16 Posterior-only, 17 anterior–posterior	78.7 ± 8.9°	51.7 ± 10.3°	49 months ± 24	1.4° ± 3.9°	Infections (3), Metallosis (4), Loss of correction (1), Rod brake (1), Proximal Junctional Kyphosis (1)
**23**	T de Jong et al., 2001, Hungary [41]	8	19 (13–27)	Cotrel–Dubousset, Posterior-only	86° (71–99°)	44° (32–58°)	60 months	4.6° (1–12°)	Superficial infection (1), Proximal loosening (1), Proximal Junctional Kyphosis (1)
**24**	Lim M et al., 2001, USA [42]	23	19	ISOLA 14, 9 Cotrel–Dubousset, 20 anterior–posterior, 3 Posterior-only	83° (63–104°)	46° (32–67°)	38 months (10–123)	N/A	Pleural effusions (7), Pneumothoraxes (2), Healing elongation (1), Arms pain (1), Loss of fixation (3)
**25**	Lowe et al., 1994, USA [43]	32	25.8 (14.3–57.6)	Cotrel–Dubousset, 28 Anterior–posterior 4 Posterior-only	85° (75–105°)	43° (26–65°)	42 months (24–74)	4° (0–19°)	Proximal Junctional Kyphosis (10), Distal Junctional Kyphosis (9), Cervical pain (4), Back pain (28)
**26**	Sturm P et al., 1992, Canada [44]	39	19 (12–37)	Harrington rod, Posterior-only	71.5°	37.7°	71.8 (23–144)	6	Intraoperative hook failure (4), Superficial wound infection (2), Retained piece of drain (1), Deep infection (1), Hook pullout (1), Broken rods (3)
**27**	Otsuka NY et al., 1990, Canada [45]	10	N/A	Harrington rod, Posterior-only	71.4°	39.3°	26.6	7.8	N/A
**28**	Herndon WA et al., 1981, USA [46]	13	19 (14–30)	Harrington rod Anterior–posterior	78°	38°	29 months (12–66)	7.8	Death (1), Laminae fracture (1), Deep Venous Thrombosis (1), Prominent hardware-revision (1)
**29**	Taylor TC et al., 1979, USA [47]	27	N/A	Harrington rod Posterior-only	72°	46.1°	27.6	5.7	N/A
**30**	Bradford DS et al., 1975, USA [48]	22	N/A	Harrington rod, Posterior-only	72°	47°	35 months	21 in 16 (72%) pts	N/A

Regarding the distal end of the fusion, Dubousset and Guillaumat in 1987 suggested that the most distal vertebra included should be the one just above the horizontal disc as noted in the hyperextension radiograph [77]. Lowe and Kasten suggested that the fusion construct should include the first lordotic disc, a finding which was supported by Denis et al. [43,89]. In the study of Cho et al. the lowest instrumented vertebra (LIV) was described as the fusion-included sagittal stable vertebra (SSV), and it was correlated with low incidence of DJK [35]. Although Baymurat et al. for the LIV usually selected the first lordotic segment, the fusion of SSV in a group of 37 patients for SK operative correction, did not display any statistical differences in the frequency of the development of DJK [25]. These results are in agreement with the study Luzzi et al. that did not observe any remarkable alteration in the rate of DJK when compared with the LIV fusion of the first lordotic vertebrae and SSV [96]. The above finding was explained by the existence of considerable overlap at the anatomical location SSV and 1st lordotic vertebrae [95]. This is consistent with the results of Jensch et al. that reported a 0% rate of DJK after the selection of the stable sagittal vertebra for the end of distal fusion [23]. Additionally, LIV fusion to two vertebrae below the first lordotic disc was correlated with significant reduced risk of DJK [97]. This, however, also remains the subject of ongoing deliberation.

Nevertheless, our review study has several limitations. First, most of the available evidence comes from single-centre retrospective studies, which often suffer from heterogeneous methodologies, variable inclusion criteria, inconsistent diagnostic definitions, and a lack of control groups. Moreover, the evaluation of the postoperative outcomes was mainly performed by imaging techniques and not by functional or clinical criteria such as pain, satisfaction or quality of life. Finally, data regarding which patients with SK are at greater risk of deformity progression is not readily available. Indicatively, in the study by Sachs et al., only 4 out of 14 patients with a kyphotic curve greater than 75 degrees required surgical treatment [55].

Future directions for the improvement of the operative techniques will include the incorporation of innovative tissue engineering discoveries such as robotics, three- or four-dimensional printing to produce individualized smart shape-adaptation instrumentations, or drug-delivering hydrogels to enhance spinal fusion and to decrease the postoperative loss of correction and infectious or neurological complications [98].

## 6. Conclusions

Our study reports the results of the up-to-date literature concerning the indications and outcomes of surgical interventions for patients with SK. Although the optimal surgical intervention has not yet been clarified, our analysis demonstrated that the recent trend towards the posterior-only approach associated with Ponte’s osteotomies provided a superior rate of correction, increased scores of patients’ satisfaction and reduced risk of complications when compared with the anterior-only approach. Moreover, the fusion levels as well as pre- and post-spinopelvic parameters focusing on lumbopelvic mismatch should be carefully considered. Notably, thoracic kyphosis correction should be correlated with preoperative pelvic incidence and tilt measurements to avoid the development of junctional kyphotic deformities. Although surgical treatment remains a reliable method for addressing kyphotic deformity, it should be approached on an individualized basis, given the complications that may arise. Future clinical studies are deemed necessary to be critical in refining patient selection criteria and optimizing surgical algorithms.

## Data Availability

Data available upon request from the authors.
